# 2,7-Bis(2,6-diisopropyl­phen­yl)benzo[*lmn*][3,8]phenanthroline-1,3,6,8(2*H*,7*H*)-tetra­one

**DOI:** 10.1107/S1600536811045065

**Published:** 2011-11-05

**Authors:** Xiang-Xiang Wu, Shu-Ling Zhang, Seik Weng Ng

**Affiliations:** a Henan University of Traditional Chinese Medicine, Zhengzhou 450008, People’s Republic of China; b Department of Chemistry, University of Malaya, 50603 Kuala Lumpur, Malaysia; c Chemistry Department, Faculty of Science, King Abdulaziz University, PO Box 80203 Jeddah, Saudi Arabia

## Abstract

In the title compound, C_38_H_38_N_2_O_4_, the 16 atoms comprising the four six-membered rings that are fused together are approximately coplanar (maximum r.m.s. deviation = 0.033 Å). The benzene rings at either ends are nearly perpendicular to the mean plane of the fused-ring system; one is aligned at 80.4 (1) ° and the other at 82.2 (1)°.

## Related literature

The title compound is the precusor to the class of 1,6-di[(trimethyl­sil­yl)ethyn­yl]naphthelene diimides. For background to this class of compound, see: Weil *et al.* (2002 ▶); Yue *et al.* (2010 ▶).
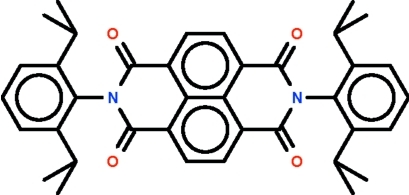


## Experimental

### 

#### Crystal data

C_38_H_38_N_2_O_4_
*M* *_r_* = 586.70
Orthorhombic, 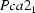

*a* = 15.9987 (13) Å
*b* = 8.560 (2) Å
*c* = 22.951 (3) Å
*V* = 3142.9 (10) Å^3^
*Z* = 4
Mo *K*α radiation
μ = 0.08 mm^−1^
*T* = 173 K
0.20 × 0.17 × 0.12 mm

#### Data collection

Rigaku Saturn724+ CCD diffractometer
Absorption correction: multi-scan (*CrystalClear*; Rigaku, 2007 ▶) *T*_min_ = 0.984, *T*_max_ = 0.990
11798 measured reflections
3684 independent reflections
3401 reflections with *I* > 2sσ(*I*)
*R*_int_ = 0.048

#### Refinement

*R*[*F* ^2^ > 2σ(*F* ^2^)] = 0.058
*wR*(*F* ^2^) = 0.144
*S* = 1.10
3684 reflections
405 parameters
1 restraint
H-atom parameters constrained
Δρ_max_ = 0.34 e Å^−3^
Δρ_min_ = −0.17 e Å^−3^

### 

Data collection: *CrystalClear* (Rigaku, 2007 ▶); cell refinement: *CrystalClear*; data reduction: *CrystalClear*; program(s) used to solve structure: *SHELXS97* (Sheldrick, 2008 ▶); program(s) used to refine structure: *SHELXL97* (Sheldrick, 2008 ▶); molecular graphics: *X-SEED* (Barbour, 2001 ▶); software used to prepare material for publication: *publCIF* (Westrip, 2010 ▶).

## Supplementary Material

Crystal structure: contains datablock(s) global, I. DOI: 10.1107/S1600536811045065/bt5694sup1.cif

Structure factors: contains datablock(s) I. DOI: 10.1107/S1600536811045065/bt5694Isup2.hkl

Supplementary material file. DOI: 10.1107/S1600536811045065/bt5694Isup3.cml

Additional supplementary materials:  crystallographic information; 3D view; checkCIF report

## Figures and Tables

**Fig. 1.**
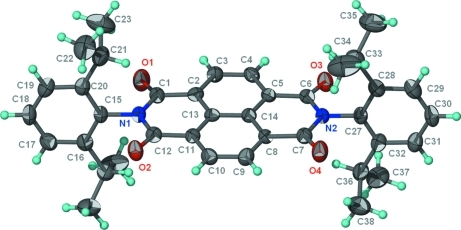
Anisotropic displacement ellipsoid plot (Barbour, 2001) of C38H38N2O4 at the 50% probability level; hydrogen atoms are drawn as spheres of arbitrary radius.

## supplementary crystallographic information

### Comment

The 2,7-bis(2,6-diisopropylphenyl)benzo[*lmn*][3,8]phenanthroline-1,3,6,8(2*H*,7*H*)-tetraone (Scheme I) that carries a trimethylsilylethynyl subsituent is the precusor to the class of 1,6-di((trimethylsilyl)ethynyl)naphthelene diimides, which are synthesized in a one-pot oxidative-coupling synthesis (Yue *et al.*, 2010). The compounds are of tremendous potential as chemosensors and photonic materials (Weil *et al.*, 2002). In the unsubstituted compound, the sixteen atoms comprising the four six-membered rings that are fused together are co-planar (r.m.s. deviation 0.033 Å); the benzene rings at either ends are perpendicular to the mean plane of the fused ring. One is aligned at 80.4 (1) ° and the other at 82.2 (1)° (Fig. 1).

### Experimental

Naphthalene-1,4,5,8-tetracarboxylic acid dianhydride (1.00 g, 3.73 mmol), 1*H*-pyrazole (2.55 g, 37.3 mmol) and 2,6-diisopropylaniline (2.65 g, 14.9 mmol) were mixed in a 100 ml flask. The mixture was heated with stirring at 393 K for 3 days. Then, the mixture was poured into dilute hydrochloric acid (1.5 *M*) to precipitate a white solid. This was dried and purified by column chromatography (petroleum ether/acetone 10:1) to yield a powder (0.92 g, 42%). Crystals were obtained by recrystallization from a 1:1 mixture of chloroform and *n*-hexane.

### Refinement

Carbon-bound H-atoms were placed in calculated positions [C–H 0.95–0.98 Å, *U*_iso_(H) 1.2–1.5*U*_eq_(C)] and were included in the refinement in the riding model approximation. Due to the absence of anomalous scatterers, 2913 Friedel pairs were merged.

### Crystal data

**Table:**

C_38_H_38_N_2_O_4_	*F*(000) = 1248
*M**_r_* = 586.70	*D*_x_ = 1.240 Mg m^−^^3^
Orthorhombic, *P**c**a*2_1_	Mo *K*α radiation, λ = 0.71073 Å
Hall symbol: P 2c -2ac	Cell parameters from 8525 reflections
*a* = 15.9987 (13) Å	θ = 1.3–27.5°
*b* = 8.560 (2) Å	µ = 0.08 mm^−^^1^
*c* = 22.951 (3) Å	*T* = 173 K
*V* = 3142.9 (10) Å^3^	Prism, colorless
*Z* = 4	0.20 × 0.17 × 0.12 mm

### Data collection

**Table:**

Rigaku Saturn724+ CCD diffractometer	3684 independent reflections
Radiation source: fine-focus sealed tube	3401 reflections with *I* > 2sσ(*I*)
graphite	*R*_int_ = 0.048
ω scans at fixed χ = 45°	θ_max_ = 27.5°, θ_min_ = 2.4°
Absorption correction: multi-scan (*CrystalClear*; Rigaku, 2007)	*h* = −16→20
*T*_min_ = 0.984, *T*_max_ = 0.990	*k* = −5→11
11798 measured reflections	*l* = −28→29

### Refinement

**Table:**

Refinement on *F*^2^	Primary atom site location: structure-invariant direct methods
Least-squares matrix: full	Secondary atom site location: difference Fourier map
*R*[*F*^2^ > 2σ(*F*^2^)] = 0.058	Hydrogen site location: inferred from neighbouring sites
*wR*(*F*^2^) = 0.144	H-atom parameters constrained
*S* = 1.10	*w* = 1/[σ^2^(*F*_o_^2^) + (0.0655*P*)^2^ + 0.7882*P*] where *P* = (*F*_o_^2^ + 2*F*_c_^2^)/3
3684 reflections	(Δ/σ)_max_ = 0.001
405 parameters	Δρ_max_ = 0.34 e Å^−^^3^
1 restraint	Δρ_min_ = −0.17 e Å^−^^3^

### Fractional atomic coordinates and isotropic or equivalent isotropic displacement parameters (Å^2^)

**Table:**

	*x*	*y*	*z*	*U*_iso_*/*U*_eq_	
O1	−0.09057 (16)	0.0036 (3)	0.50005 (14)	0.0454 (7)	
O2	0.11892 (15)	0.0356 (3)	0.63278 (10)	0.0356 (6)	
O3	0.13901 (16)	0.4806 (3)	0.30735 (11)	0.0368 (6)	
O4	0.34787 (16)	0.5169 (3)	0.44096 (13)	0.0424 (7)	
N1	0.01330 (16)	0.0239 (3)	0.56671 (13)	0.0272 (6)	
N2	0.24226 (17)	0.5012 (3)	0.37466 (13)	0.0267 (6)	
C1	−0.0234 (2)	0.0588 (4)	0.51296 (15)	0.0302 (7)	
C2	0.02342 (19)	0.1660 (4)	0.47403 (15)	0.0276 (7)	
C3	−0.0107 (2)	0.2127 (4)	0.42239 (15)	0.0319 (7)	
H3	−0.0659	0.1814	0.4126	0.038*	
C4	0.0350 (2)	0.3067 (4)	0.38356 (15)	0.0293 (7)	
H4	0.0108	0.3376	0.3476	0.035*	
C5	0.11502 (19)	0.3544 (3)	0.39758 (14)	0.0255 (6)	
C6	0.16344 (19)	0.4481 (3)	0.35570 (14)	0.0261 (6)	
C7	0.2800 (2)	0.4643 (4)	0.42800 (15)	0.0302 (7)	
C8	0.23275 (19)	0.3582 (4)	0.46743 (14)	0.0268 (6)	
C9	0.2674 (2)	0.3101 (4)	0.51908 (16)	0.0321 (7)	
H9	0.3225	0.3415	0.5291	0.038*	
C10	0.2216 (2)	0.2143 (4)	0.55734 (15)	0.0313 (7)	
H10	0.2460	0.1809	0.5929	0.038*	
C11	0.14161 (19)	0.1689 (3)	0.54343 (14)	0.0255 (6)	
C12	0.0926 (2)	0.0731 (4)	0.58520 (14)	0.0291 (7)	
C13	0.10467 (19)	0.2143 (4)	0.49000 (14)	0.0247 (6)	
C14	0.15048 (19)	0.3097 (4)	0.45142 (14)	0.0255 (6)	
C15	−0.03363 (19)	−0.0757 (4)	0.60631 (14)	0.0282 (6)	
C16	−0.0153 (2)	−0.2354 (4)	0.60620 (15)	0.0321 (7)	
C17	−0.0608 (2)	−0.3277 (4)	0.64493 (18)	0.0378 (8)	
H17	−0.0502	−0.4368	0.6465	0.045*	
C18	−0.1210 (2)	−0.2640 (4)	0.68110 (16)	0.0381 (8)	
H18	−0.1510	−0.3293	0.7073	0.046*	
C19	−0.1377 (2)	−0.1061 (4)	0.67923 (16)	0.0365 (8)	
H19	−0.1798	−0.0642	0.7039	0.044*	
C20	−0.0944 (2)	−0.0066 (4)	0.64205 (16)	0.0302 (7)	
C21	−0.1142 (2)	0.1665 (4)	0.63976 (18)	0.0411 (9)	
H21	−0.0682	0.2193	0.6178	0.049*	
C22	−0.1180 (3)	0.2383 (5)	0.6997 (2)	0.0590 (12)	
H22C	−0.1683	0.2016	0.7198	0.089*	
H22B	−0.1199	0.3524	0.6962	0.089*	
H22A	−0.0684	0.2077	0.7219	0.089*	
C23	−0.1964 (3)	0.1969 (5)	0.6067 (3)	0.0701 (15)	
H23A	−0.1936	0.1479	0.5681	0.105*	
H23B	−0.2047	0.3097	0.6022	0.105*	
H23C	−0.2432	0.1524	0.6286	0.105*	
C24	0.0508 (2)	−0.3048 (4)	0.56679 (18)	0.0379 (8)	
H24	0.0904	−0.2184	0.5569	0.046*	
C25	0.0129 (3)	−0.3612 (6)	0.5094 (2)	0.0628 (13)	
H25C	−0.0220	−0.4532	0.5167	0.094*	
H25B	0.0578	−0.3888	0.4823	0.094*	
H25A	−0.0213	−0.2779	0.4925	0.094*	
C26	0.1022 (3)	−0.4323 (5)	0.5957 (2)	0.0542 (11)	
H26C	0.0656	−0.5194	0.6064	0.081*	
H26B	0.1290	−0.3904	0.6307	0.081*	
H26A	0.1451	−0.4692	0.5685	0.081*	
C27	0.28894 (19)	0.6031 (4)	0.33543 (14)	0.0279 (7)	
C28	0.2721 (2)	0.7625 (4)	0.33621 (16)	0.0336 (7)	
C29	0.3192 (2)	0.8567 (4)	0.29950 (17)	0.0403 (9)	
H29	0.3099	0.9663	0.2992	0.048*	
C30	0.3792 (2)	0.7935 (4)	0.26349 (17)	0.0381 (8)	
H30	0.4114	0.8601	0.2391	0.046*	
C31	0.3930 (2)	0.6345 (4)	0.26247 (15)	0.0355 (8)	
H31	0.4339	0.5926	0.2368	0.043*	
C32	0.3477 (2)	0.5341 (4)	0.29866 (17)	0.0327 (8)	
C33	0.2047 (3)	0.8297 (4)	0.37624 (18)	0.0408 (9)	
H33	0.1631	0.7444	0.3830	0.049*	
C34	0.2384 (4)	0.8743 (7)	0.4345 (2)	0.0814 (18)	
H34A	0.2717	0.7879	0.4502	0.122*	
H34B	0.1920	0.8971	0.4610	0.122*	
H34C	0.2737	0.9672	0.4305	0.122*	
C35	0.1569 (3)	0.9658 (6)	0.3493 (2)	0.0679 (14)	
H35C	0.1935	1.0573	0.3468	0.102*	
H35A	0.1083	0.9907	0.3735	0.102*	
H35B	0.1381	0.9368	0.3101	0.102*	
C36	0.3629 (2)	0.3581 (4)	0.29656 (18)	0.0382 (8)	
H36	0.3154	0.3068	0.3174	0.046*	
C37	0.3632 (3)	0.2961 (5)	0.2343 (2)	0.0522 (11)	
H37B	0.3110	0.3259	0.2149	0.078*	
H37A	0.3680	0.1820	0.2350	0.078*	
H37C	0.4107	0.3406	0.2131	0.078*	
C38	0.4432 (3)	0.3124 (5)	0.3284 (2)	0.0550 (11)	
H38C	0.4914	0.3575	0.3081	0.083*	
H38B	0.4484	0.1983	0.3290	0.083*	
H38A	0.4413	0.3519	0.3684	0.083*	

### Atomic displacement parameters (Å^2^)

**Table:**

	*U*^11^	*U*^22^	*U*^33^	*U*^12^	*U*^13^	*U*^23^
O1	0.0350 (13)	0.0568 (16)	0.0444 (17)	−0.0158 (12)	−0.0071 (14)	0.0115 (13)
O2	0.0417 (13)	0.0404 (13)	0.0248 (13)	−0.0083 (10)	−0.0029 (12)	0.0050 (10)
O3	0.0400 (13)	0.0417 (14)	0.0288 (14)	−0.0063 (11)	−0.0023 (12)	0.0061 (10)
O4	0.0326 (13)	0.0556 (16)	0.0390 (16)	−0.0150 (12)	−0.0062 (12)	0.0117 (12)
N1	0.0280 (13)	0.0274 (13)	0.0261 (15)	−0.0022 (10)	0.0024 (13)	0.0018 (11)
N2	0.0273 (13)	0.0263 (12)	0.0263 (14)	−0.0007 (10)	0.0019 (12)	0.0037 (10)
C1	0.0297 (15)	0.0318 (16)	0.0292 (17)	−0.0001 (13)	−0.0004 (14)	0.0045 (14)
C2	0.0235 (15)	0.0276 (15)	0.0318 (17)	0.0016 (12)	−0.0010 (13)	−0.0017 (13)
C3	0.0265 (15)	0.0365 (17)	0.0327 (18)	−0.0006 (13)	−0.0029 (14)	0.0041 (14)
C4	0.0277 (16)	0.0290 (15)	0.0313 (18)	−0.0006 (12)	−0.0064 (13)	0.0053 (13)
C5	0.0264 (14)	0.0237 (14)	0.0264 (16)	0.0026 (12)	−0.0006 (13)	0.0014 (11)
C6	0.0284 (14)	0.0245 (14)	0.0252 (16)	0.0017 (12)	−0.0012 (13)	−0.0002 (12)
C7	0.0289 (16)	0.0310 (16)	0.0308 (18)	−0.0011 (13)	−0.0008 (14)	0.0021 (13)
C8	0.0263 (15)	0.0255 (15)	0.0285 (16)	0.0009 (12)	0.0004 (13)	0.0037 (12)
C9	0.0249 (15)	0.0382 (17)	0.0331 (18)	−0.0074 (13)	−0.0032 (13)	0.0004 (14)
C10	0.0317 (15)	0.0340 (16)	0.0283 (18)	−0.0009 (13)	−0.0061 (14)	0.0033 (13)
C11	0.0271 (14)	0.0257 (14)	0.0238 (15)	0.0014 (12)	0.0009 (13)	−0.0018 (12)
C12	0.0312 (15)	0.0280 (15)	0.0282 (17)	0.0005 (13)	−0.0005 (14)	−0.0017 (12)
C13	0.0244 (14)	0.0241 (13)	0.0255 (15)	0.0043 (11)	0.0000 (12)	−0.0016 (12)
C14	0.0257 (14)	0.0243 (14)	0.0266 (16)	0.0010 (11)	0.0004 (13)	−0.0005 (11)
C15	0.0288 (15)	0.0310 (15)	0.0247 (15)	−0.0042 (12)	0.0008 (13)	0.0025 (13)
C16	0.0316 (16)	0.0331 (16)	0.0315 (17)	−0.0029 (13)	−0.0037 (15)	0.0021 (14)
C17	0.0379 (17)	0.0294 (16)	0.046 (2)	−0.0020 (14)	−0.0031 (17)	0.0054 (15)
C18	0.0373 (17)	0.045 (2)	0.0318 (18)	−0.0108 (16)	0.0020 (16)	0.0103 (16)
C19	0.0331 (17)	0.0439 (19)	0.0325 (18)	−0.0066 (15)	0.0045 (16)	−0.0052 (15)
C20	0.0297 (15)	0.0335 (16)	0.0273 (18)	−0.0051 (13)	−0.0005 (14)	−0.0005 (13)
C21	0.0437 (19)	0.0350 (18)	0.044 (2)	−0.0036 (15)	0.0143 (18)	−0.0054 (15)
C22	0.071 (3)	0.048 (2)	0.058 (3)	−0.008 (2)	0.014 (2)	−0.015 (2)
C23	0.073 (3)	0.046 (2)	0.091 (4)	0.020 (2)	−0.016 (3)	−0.001 (2)
C24	0.0396 (18)	0.0324 (16)	0.042 (2)	0.0002 (14)	0.0045 (17)	−0.0011 (15)
C25	0.057 (2)	0.081 (3)	0.050 (3)	0.023 (2)	−0.008 (2)	−0.027 (2)
C26	0.051 (2)	0.047 (2)	0.065 (3)	0.0150 (19)	0.002 (2)	0.001 (2)
C27	0.0289 (15)	0.0285 (15)	0.0261 (16)	−0.0065 (12)	−0.0011 (13)	0.0025 (12)
C28	0.0380 (17)	0.0284 (15)	0.0343 (18)	−0.0013 (13)	−0.0024 (16)	0.0018 (13)
C29	0.050 (2)	0.0273 (16)	0.044 (2)	−0.0032 (15)	−0.0018 (18)	0.0056 (15)
C30	0.0442 (19)	0.0352 (18)	0.0349 (19)	−0.0141 (15)	−0.0017 (17)	0.0076 (14)
C31	0.0372 (18)	0.0430 (19)	0.0263 (17)	−0.0053 (15)	0.0033 (15)	0.0015 (14)
C32	0.0315 (17)	0.0335 (17)	0.033 (2)	−0.0061 (14)	0.0042 (16)	−0.0024 (14)
C33	0.048 (2)	0.0289 (16)	0.045 (2)	0.0058 (15)	0.0038 (18)	−0.0013 (15)
C34	0.072 (3)	0.111 (4)	0.062 (3)	0.033 (3)	−0.015 (3)	−0.031 (3)
C35	0.082 (3)	0.064 (3)	0.058 (3)	0.036 (3)	0.001 (3)	−0.002 (2)
C36	0.0393 (18)	0.0304 (17)	0.045 (2)	−0.0022 (14)	0.0118 (17)	0.0002 (15)
C37	0.053 (2)	0.051 (2)	0.052 (3)	0.0014 (19)	0.005 (2)	−0.0140 (19)
C38	0.061 (3)	0.043 (2)	0.062 (3)	0.014 (2)	0.000 (2)	0.001 (2)

### Geometric parameters (Å, °)

**Table:**

O1—C1	1.211 (4)	C22—H22B	0.9800
O2—C12	1.213 (4)	C22—H22A	0.9800
O3—C6	1.209 (4)	C23—H23A	0.9800
O4—C7	1.213 (4)	C23—H23B	0.9800
N1—C1	1.399 (4)	C23—H23C	0.9800
N1—C12	1.403 (4)	C24—C26	1.518 (5)
N1—C15	1.455 (4)	C24—C25	1.528 (6)
N2—C7	1.401 (4)	C24—H24	1.0000
N2—C6	1.409 (4)	C25—H25C	0.9800
N2—C27	1.459 (4)	C25—H25B	0.9800
C1—C2	1.483 (4)	C25—H25A	0.9800
C2—C3	1.365 (5)	C26—H26C	0.9800
C2—C13	1.413 (4)	C26—H26B	0.9800
C3—C4	1.406 (5)	C26—H26A	0.9800
C3—H3	0.9500	C27—C28	1.391 (5)
C4—C5	1.381 (4)	C27—C32	1.394 (5)
C4—H4	0.9500	C28—C29	1.389 (5)
C5—C14	1.413 (5)	C28—C33	1.529 (5)
C5—C6	1.472 (4)	C29—C30	1.377 (6)
C7—C8	1.488 (4)	C29—H29	0.9500
C8—C9	1.371 (5)	C30—C31	1.379 (5)
C8—C14	1.428 (4)	C30—H30	0.9500
C9—C10	1.407 (5)	C31—C32	1.398 (5)
C9—H9	0.9500	C31—H31	0.9500
C10—C11	1.375 (4)	C32—C36	1.526 (5)
C10—H10	0.9500	C33—C34	1.492 (7)
C11—C13	1.416 (5)	C33—C35	1.525 (6)
C11—C12	1.485 (4)	C33—H33	1.0000
C13—C14	1.410 (4)	C34—H34A	0.9800
C15—C16	1.398 (4)	C34—H34B	0.9800
C15—C20	1.403 (5)	C34—H34C	0.9800
C16—C17	1.395 (5)	C35—H35C	0.9800
C16—C24	1.514 (5)	C35—H35A	0.9800
C17—C18	1.384 (5)	C35—H35B	0.9800
C17—H17	0.9500	C36—C37	1.524 (6)
C18—C19	1.379 (5)	C36—C38	1.530 (6)
C18—H18	0.9500	C36—H36	1.0000
C19—C20	1.390 (5)	C37—H37B	0.9800
C19—H19	0.9500	C37—H37A	0.9800
C20—C21	1.517 (5)	C37—H37C	0.9800
C21—C22	1.507 (6)	C38—H38C	0.9800
C21—C23	1.541 (6)	C38—H38B	0.9800
C21—H21	1.0000	C38—H38A	0.9800
C22—H22C	0.9800		
			
C1—N1—C12	125.6 (3)	C21—C23—H23A	109.5
C1—N1—C15	117.4 (3)	C21—C23—H23B	109.5
C12—N1—C15	117.0 (3)	H23A—C23—H23B	109.5
C7—N2—C6	125.6 (3)	C21—C23—H23C	109.5
C7—N2—C27	117.0 (3)	H23A—C23—H23C	109.5
C6—N2—C27	117.4 (3)	H23B—C23—H23C	109.5
O1—C1—N1	120.3 (3)	C16—C24—C26	113.6 (3)
O1—C1—C2	122.8 (3)	C16—C24—C25	111.2 (3)
N1—C1—C2	116.8 (3)	C26—C24—C25	111.3 (3)
C3—C2—C13	120.5 (3)	C16—C24—H24	106.8
C3—C2—C1	120.2 (3)	C26—C24—H24	106.8
C13—C2—C1	119.3 (3)	C25—C24—H24	106.8
C2—C3—C4	120.7 (3)	C24—C25—H25C	109.5
C2—C3—H3	119.7	C24—C25—H25B	109.5
C4—C3—H3	119.7	H25C—C25—H25B	109.5
C5—C4—C3	120.2 (3)	C24—C25—H25A	109.5
C5—C4—H4	119.9	H25C—C25—H25A	109.5
C3—C4—H4	119.9	H25B—C25—H25A	109.5
C4—C5—C14	119.7 (3)	C24—C26—H26C	109.5
C4—C5—C6	119.8 (3)	C24—C26—H26B	109.5
C14—C5—C6	120.5 (3)	H26C—C26—H26B	109.5
O3—C6—N2	119.9 (3)	C24—C26—H26A	109.5
O3—C6—C5	123.7 (3)	H26C—C26—H26A	109.5
N2—C6—C5	116.4 (3)	H26B—C26—H26A	109.5
O4—C7—N2	121.1 (3)	C28—C27—C32	123.6 (3)
O4—C7—C8	122.2 (3)	C28—C27—N2	118.6 (3)
N2—C7—C8	116.8 (3)	C32—C27—N2	117.7 (3)
C9—C8—C14	120.5 (3)	C29—C28—C27	117.2 (3)
C9—C8—C7	120.2 (3)	C29—C28—C33	121.9 (3)
C14—C8—C7	119.3 (3)	C27—C28—C33	120.9 (3)
C8—C9—C10	120.3 (3)	C30—C29—C28	121.0 (3)
C8—C9—H9	119.9	C30—C29—H29	119.5
C10—C9—H9	119.9	C28—C29—H29	119.5
C11—C10—C9	120.3 (3)	C31—C30—C29	120.6 (3)
C11—C10—H10	119.9	C31—C30—H30	119.7
C9—C10—H10	119.9	C29—C30—H30	119.7
C10—C11—C13	120.8 (3)	C30—C31—C32	120.9 (3)
C10—C11—C12	119.8 (3)	C30—C31—H31	119.5
C13—C11—C12	119.4 (3)	C32—C31—H31	119.5
O2—C12—N1	120.4 (3)	C27—C32—C31	116.7 (3)
O2—C12—C11	123.0 (3)	C27—C32—C36	123.0 (3)
N1—C12—C11	116.6 (3)	C31—C32—C36	120.3 (3)
C14—C13—C2	119.0 (3)	C34—C33—C35	110.5 (4)
C14—C13—C11	119.1 (3)	C34—C33—C28	112.3 (4)
C2—C13—C11	121.9 (3)	C35—C33—C28	113.4 (3)
C13—C14—C5	119.8 (3)	C34—C33—H33	106.7
C13—C14—C8	119.1 (3)	C35—C33—H33	106.7
C5—C14—C8	121.1 (3)	C28—C33—H33	106.7
C16—C15—C20	123.9 (3)	C33—C34—H34A	109.5
C16—C15—N1	117.6 (3)	C33—C34—H34B	109.5
C20—C15—N1	118.4 (3)	H34A—C34—H34B	109.5
C15—C16—C17	116.3 (3)	C33—C34—H34C	109.5
C15—C16—C24	122.1 (3)	H34A—C34—H34C	109.5
C17—C16—C24	121.6 (3)	H34B—C34—H34C	109.5
C18—C17—C16	121.5 (3)	C33—C35—H35C	109.5
C18—C17—H17	119.2	C33—C35—H35A	109.5
C16—C17—H17	119.2	H35C—C35—H35A	109.5
C19—C18—C17	120.1 (3)	C33—C35—H35B	109.5
C19—C18—H18	119.9	H35C—C35—H35B	109.5
C17—C18—H18	119.9	H35A—C35—H35B	109.5
C18—C19—C20	121.6 (3)	C37—C36—C32	112.0 (3)
C18—C19—H19	119.2	C37—C36—C38	110.9 (3)
C20—C19—H19	119.2	C32—C36—C38	111.8 (3)
C19—C20—C15	116.5 (3)	C37—C36—H36	107.3
C19—C20—C21	121.1 (3)	C32—C36—H36	107.3
C15—C20—C21	122.4 (3)	C38—C36—H36	107.3
C22—C21—C20	112.0 (4)	C36—C37—H37B	109.5
C22—C21—C23	110.2 (4)	C36—C37—H37A	109.5
C20—C21—C23	111.1 (3)	H37B—C37—H37A	109.5
C22—C21—H21	107.8	C36—C37—H37C	109.5
C20—C21—H21	107.8	H37B—C37—H37C	109.5
C23—C21—H21	107.8	H37A—C37—H37C	109.5
C21—C22—H22C	109.5	C36—C38—H38C	109.5
C21—C22—H22B	109.5	C36—C38—H38B	109.5
H22C—C22—H22B	109.5	H38C—C38—H38B	109.5
C21—C22—H22A	109.5	C36—C38—H38A	109.5
H22C—C22—H22A	109.5	H38C—C38—H38A	109.5
H22B—C22—H22A	109.5	H38B—C38—H38A	109.5
			
C12—N1—C1—O1	176.1 (3)	C6—C5—C14—C8	2.1 (4)
C15—N1—C1—O1	−2.0 (5)	C9—C8—C14—C13	1.3 (5)
C12—N1—C1—C2	−4.4 (5)	C7—C8—C14—C13	−177.4 (3)
C15—N1—C1—C2	177.4 (3)	C9—C8—C14—C5	−178.1 (3)
O1—C1—C2—C3	3.3 (5)	C7—C8—C14—C5	3.2 (4)
N1—C1—C2—C3	−176.1 (3)	C1—N1—C15—C16	96.0 (3)
O1—C1—C2—C13	−174.4 (3)	C12—N1—C15—C16	−82.3 (4)
N1—C1—C2—C13	6.2 (4)	C1—N1—C15—C20	−83.8 (4)
C13—C2—C3—C4	1.5 (5)	C12—N1—C15—C20	97.9 (3)
C1—C2—C3—C4	−176.2 (3)	C20—C15—C16—C17	−0.7 (5)
C2—C3—C4—C5	−0.7 (5)	N1—C15—C16—C17	179.5 (3)
C3—C4—C5—C14	−0.7 (5)	C20—C15—C16—C24	179.9 (3)
C3—C4—C5—C6	177.8 (3)	N1—C15—C16—C24	0.1 (5)
C7—N2—C6—O3	−175.9 (3)	C15—C16—C17—C18	0.4 (5)
C27—N2—C6—O3	3.8 (4)	C24—C16—C17—C18	179.8 (3)
C7—N2—C6—C5	4.3 (4)	C16—C17—C18—C19	0.4 (6)
C27—N2—C6—C5	−175.9 (3)	C17—C18—C19—C20	−0.8 (6)
C4—C5—C6—O3	−4.0 (5)	C18—C19—C20—C15	0.5 (5)
C14—C5—C6—O3	174.5 (3)	C18—C19—C20—C21	179.1 (3)
C4—C5—C6—N2	175.8 (3)	C16—C15—C20—C19	0.2 (5)
C14—C5—C6—N2	−5.7 (4)	N1—C15—C20—C19	−179.9 (3)
C6—N2—C7—O4	−179.2 (3)	C16—C15—C20—C21	−178.3 (3)
C27—N2—C7—O4	1.0 (5)	N1—C15—C20—C21	1.5 (5)
C6—N2—C7—C8	0.8 (4)	C19—C20—C21—C22	48.7 (5)
C27—N2—C7—C8	−179.0 (3)	C15—C20—C21—C22	−132.8 (4)
O4—C7—C8—C9	−3.4 (5)	C19—C20—C21—C23	−75.1 (5)
N2—C7—C8—C9	176.6 (3)	C15—C20—C21—C23	103.3 (4)
O4—C7—C8—C14	175.4 (3)	C15—C16—C24—C26	141.0 (4)
N2—C7—C8—C14	−4.6 (4)	C17—C16—C24—C26	−38.4 (5)
C14—C8—C9—C10	−1.0 (5)	C15—C16—C24—C25	−92.5 (4)
C7—C8—C9—C10	177.7 (3)	C17—C16—C24—C25	88.1 (4)
C8—C9—C10—C11	−0.4 (5)	C7—N2—C27—C28	−95.6 (4)
C9—C10—C11—C13	1.5 (5)	C6—N2—C27—C28	84.6 (4)
C9—C10—C11—C12	−177.6 (3)	C7—N2—C27—C32	85.0 (4)
C1—N1—C12—O2	−179.7 (3)	C6—N2—C27—C32	−94.7 (4)
C15—N1—C12—O2	−1.5 (4)	C32—C27—C28—C29	−2.4 (5)
C1—N1—C12—C11	−0.7 (4)	N2—C27—C28—C29	178.3 (3)
C15—N1—C12—C11	177.4 (3)	C32—C27—C28—C33	177.9 (3)
C10—C11—C12—O2	2.3 (5)	N2—C27—C28—C33	−1.4 (5)
C13—C11—C12—O2	−176.8 (3)	C27—C28—C29—C30	0.8 (5)
C10—C11—C12—N1	−176.6 (3)	C33—C28—C29—C30	−179.5 (3)
C13—C11—C12—N1	4.3 (4)	C28—C29—C30—C31	1.0 (6)
C3—C2—C13—C14	−0.9 (4)	C29—C30—C31—C32	−1.2 (6)
C1—C2—C13—C14	176.8 (3)	C28—C27—C32—C31	2.2 (5)
C3—C2—C13—C11	179.5 (3)	N2—C27—C32—C31	−178.5 (3)
C1—C2—C13—C11	−2.8 (4)	C28—C27—C32—C36	−177.4 (3)
C10—C11—C13—C14	−1.2 (4)	N2—C27—C32—C36	1.9 (5)
C12—C11—C13—C14	177.9 (3)	C30—C31—C32—C27	−0.3 (5)
C10—C11—C13—C2	178.4 (3)	C30—C31—C32—C36	179.3 (4)
C12—C11—C13—C2	−2.5 (4)	C29—C28—C33—C34	−89.9 (5)
C2—C13—C14—C5	−0.4 (4)	C27—C28—C33—C34	89.8 (5)
C11—C13—C14—C5	179.2 (3)	C29—C28—C33—C35	36.3 (5)
C2—C13—C14—C8	−179.8 (3)	C27—C28—C33—C35	−144.0 (4)
C11—C13—C14—C8	−0.2 (4)	C27—C32—C36—C37	131.3 (4)
C4—C5—C14—C13	1.2 (4)	C31—C32—C36—C37	−48.3 (5)
C6—C5—C14—C13	−177.3 (3)	C27—C32—C36—C38	−103.6 (4)
C4—C5—C14—C8	−179.4 (3)	C31—C32—C36—C38	76.9 (5)
